# Identification of Key Gene Networks Associated With Cell Wall Components Leading to Flesh Firmness in Watermelon

**DOI:** 10.3389/fpls.2021.630243

**Published:** 2021-06-22

**Authors:** Muhammad Anees, Lei Gao, Muhammad Jawad Umer, Pingli Yuan, Hongju Zhu, Xuqiang Lu, Nan He, Chengsheng Gong, Mohamed Omar Kaseb, Shengjie Zhao, Wenge Liu

**Affiliations:** Henan Joint International Research Laboratory of South Asian Fruits and Cucurbits, Zhengzhou Fruit Research Institute, Chinese Academy of Agricultural Sciences, Zhengzhou, China

**Keywords:** watermelon, flesh firmness, cell wall components, correlated gene-networks, WGCNA

## Abstract

Flesh firmness of watermelon is an important quality trait for commercial fruit values, including fruit storability, transportability, and shelf life. To date, knowledge of the gene networks underlying this trait is still limited. Herein, we used weighted genes co-expression network analysis (WGCNA) based on correlation and the association of phenotypic data (cell wall contents) with significantly differentially expressed genes between two materials, a near isogeneic line “HWF” (with high average flesh firmness) and inbred line “203Z” (with low average flesh firmness), to identify the gene networks responsible for changes in fruit flesh firmness. We identified three gene modules harboring 354 genes; these gene modules demonstrated significant correlation with water-soluble pectin, cellulose, hemicellulose, and protopectin. Based on intramodular significance, eight genes involved in cell wall biosynthesis and ethylene pathway are identified as hub genes within these modules. Among these genes, two genes, *Cla012351* (Cellulose synthase) and *Cla004251* (Pectinesterase), were significantly correlated with cellulose (*r*^2^ = 0.83) and protopectin (*r*^2^ = 0.81); three genes, *Cla004120* (ERF1), *Cla009966* (Cellulose synthase), and *Cla006648* (Galactosyltransferase), had a significant correlation with water-soluble pectin (*r*^2^ = 0.91), cellulose (*r*^2^ = 0.9), and protopectin (*r*^2^ = 0.92); and three genes, *Cla007092* (ERF2a), *Cla004119* (probable glycosyltransferase), and *Cla018816* (Xyloglucan endotransglucosylase/hydrolase), were correlated with hemicellulose (*r*^2^ = 0.85), cellulose (*r*^2^ = 0.8), and protopectin (*r*^2^ = 0.8). This study generated important insights of biosynthesis of a cell wall structure and ethylene signaling transduction pathway, the mechanism controlling the flesh firmness changes in watermelon, which provide a significant source to accelerate future functional analysis in watermelon to facilitate crop improvement.

## Introduction

Watermelon (*Citrullus lanatus*) is included in the genus *Citrullus* of the family Cucurbitaceae. The possible center of origin and diversity is southern Africa (Erickson et al., 2005). Watermelon constitutes 7% of the global area dedicated to vegetable production. The annual worldwide production of watermelon is about 103 million tons, making it among the top five most consumed fresh fruits^1^, and because of its sweet and juicy flavor and rich nutrition – vitamins, minerals, antioxidants, and fiber – it has become an important worldwide horticultural crop (Guo et al., 2011; Jawad et al., 2020). It produces large edible fruits that contain nutrients, including sugars, carotenoids, and lycopene, that serve as an important component in the human diet (Erickson et al., 2005). The quality of watermelon fruits is affected by many factors: fruit shape and size, rind thickness and color, flesh firmness and color, aroma, sugar contents, and carotenoids and flavonoids composition (Wechter et al., 2008). These qualities are further categorized into three main categories: commodity quality, sensory quality, and nutritional quality. Flesh firmness is the sensory quality of watermelon; the attribute is one of the important indicators to measure the commerciality, transportability, storability, and shelf life of fruits (Guo et al., 2015), the juiciness of fruits attributes to the fruit texture: if the fruit flesh is hard, the juice is low, and it provides poor mouth sensation (Harker et al., 2003). If the flesh is too soft, it will affect the degree of refreshment and shelf-life (Risse et al., 1990).

Watermelon flesh firmness change is an orderly and complex biological process involving a series of physiological and biochemical changes, including changes in cell wall contents, cell wall degradation, and other related metabolic changes (Gao et al., 2020). The changes in flesh firmness are mainly caused by changes in flesh cells’ structure and material composition. The degradation of flesh cell wall material and the destruction of pectin, cellulose, and hemicellulose structure are the beginning of the fruit softening process; a series of complex and comprehensive changes occurred in the structure of the cellulose and pectin components and the content of the intracellular material. These progressive changes lead to the degradation of cell wall polymers and loss of integrity of middle lamella (Wakabayashi, 2000; Guo et al., 2015). The earlier studies revealed that the softening of fruit is because of cell wall components; the cell wall of fruit flesh cells mainly including pectin, cellulose, hemicellulose, and alcohol-insoluble solids. Cellulose degradation leads to cell wall disintegration and fruit loss in flesh firmness (Brummell et al., 2004). The loss in flesh firmness of peach is significantly correlated with original pectin and cellulose but negatively correlated with soluble pectin content, in apple the changes in flesh firmness are due to variations in cell wall contents, pectin, cellulose, and hemicellulose, and some significant positive and negative correlations between water-soluble pectin (WSP) and hemicellulose (Qin and Xiaolin, 2007). Degradation of cell wall structural substances is affected by the expression of a series of key enzymes, mainly including polygalacturonidase (*PG*), cellulase (*Cx*), pectin methylesterase (*PME*), β-galactosidase (β-*Gal*), lipoxygenase (*LOX*), and pectin lyase. *PG* can catalyze the hydrolysis polygalacturonase in the fruit cell wall to produce galacturonic acid, which promotes the degradation of pectin, thereby reducing the low cell viscosity, resulting in softening of the fruit. With exogenous ethylene, the decrease and increase expression of Polygalacturonase genes *PpPG2, PpPG1*, pectin methylesterase genes, *PpPME1, PpPME2* in stony and soft peach involve in the solubilization of polyuronides, which cause the loss in firmness of peach (Murayama et al., 2009). To clarify the mechanisms involved in fruit softening and textural changes in pear 13 genes related to polygalacturonases (*PG*), pectin methylesterases (*PME*), α arabinofuranosidase (*ARF*), β-galactosidases (*GAL*), and endo-1,4-β-d-glucanases (*Cel*) are investigated, and results showed the *PcPG3*, *PcPME1*, *PcPME2*, *PcPME3*, *PcGAL1*, *PcGAL2*, and *PcCel2* might be involved in the loss in firmness (Sekine et al., 2006). Variation in fruit firmness is more significant in apple cultivars, and investigation of firmness related genes (*MdACS1*, *MdACO1*, *MdPG1*, and *MdExp3*) revealed that the loss in firmness might depend on the expression pattern of *MdPG1;* in addition to *MdPG1*, *Md*β-*GAL2* may play a central role in apple fruit softening (Wakasa et al., 2006; Gwanpua et al., 2016). In tomato, the QTL for fruit firmness is mapped on chromosome 2 underlying *ERF* and *PME* genes. A quantitative reverse transcription-polymerase chain reaction was used to quantify the expression: the increased expression of *ERF* is related to softening, and the *PME* expression is tightly related to the fruit firmness (Chapman et al., 2012). The Pectate lyase *SlPL* (*Solyc03g111690*) is also reported as an excellent candidate gene for improvement of fruit firmness in tomato (Yang et al., 2017). In strawberry the depolymerization and solubilization of pectin increase during fruit developments and contributed to loss in fruit firmness, the cloning and expression revealed that two *PG* genes, *FaPG1* and T-*PG*, show increase-decreased and decreased-increased expression in soft and hard cultivars, which increased the confidence that these genes are related with the firmness of fruit (Villarreal et al., 2008).

Advances in next-generation sequencing technologies has driven DNA sequencing costs and narrowed the finding QTLs to be quick, simple, and specific regions underlying the main gene(s). In melon while working on the diverse melon horticultural groups and identified very important QTLs on chromosomes 6, 8, 9, 11, and 12 with high, moderate stringency to fruit flesh firmness of melon, using both genome-wide association studies (GWAS) and biparental mapping (Nimmakayala et al., 2016) by comparing the PG sequence of “Fuji” (Fj) and “Mondial Gala” (MG), two apple cultivars. Also identified were the PG-ethylene-related gene on chromosome 10, which controls fruit flesh firmness and ripening of apple (Fabrizio et al., 2010). A previous study on tomato fine mapped the fruit flesh firmness related genes in a physical interval of 8.6 Mb on chromosome 2; in tomato, the QTL consists of five peaks. After further clarification, ethylene response factor (ERF) underlying Firs.p.QTL2.2 and a region containing three pectin methylesterase (PME) genes underlying Firs.p.QTL2.5 was nominated as the main QTL controlling flesh firmness (Chapman et al., 2012). However, very little information is currently available at the molecular level regarding fruit flesh firmness of watermelon, a non-climacteric and economically important fruit. Herein, we generated RNA sequencing data and quantified cell wall contents, water-soluble pectin (WSP), ionic bond pectin (ISP), protopectin, cellulose, and hemicellulose in HWF a near isogenic line with high average flesh firmness and an inbred line 203Z with low average flesh firmness at 10, 18, 26, and 34 days after pollination (DAP), to reveal the regulatory networks and mechanisms controlling fruit flesh firmness in watermelon. In analyses of transcriptome data and co-expression networks analyses (WGCNA), we identified the gene-networks linked to the regulatory mechanism of fruit flesh firmness in watermelon.

## Materials and Methods

### Plant Material

Experimental materials included “HWF”, a near-isogenic line (NIL) containing exotic segments from the wild watermelon “PI271769” and the recurrent parent “203Z”. “203Z” and “PI271769” used as germplasm resources were conserved in Zhengzhou Fruit Research Institute, CAAS, China. A detailed description of germplasm is also available in our previous study (Umer et al., 2020). The inbred line “203Z” has spherical fruits with a green rind, dark green stripes, red flesh, and average flesh firmness up to (1.0 kg/cm^2^) at maturity. “PI271769” belongs to the watermelon species *Citrullus amarus* and has spherical fruits with white flesh color and average flesh firmness up to (5.7 kg/cm^2^). The near-isogenic line “HWF” was derived from a cross between “203Z” and “PI271769”. The offspring were selected based on the phenotypic evaluation. Plants with high flesh firmness were backcrossed seven times using “203Z” as a recurrent parent to generate BC_7_F_1_, which is then self-pollinated four times to yield BC_7_F_5_. After phenotypic evaluation, the progeny with stable high flesh firmness was selected and named “HWF”. The experimental materials were grown in a research base located in Xinxiang, Henan province, China. Experimental design was a randomized complete block design (RCBD), with ten plants in each row, and a plant to plant distance of 50 cm was maintained. The female flowers were manually self-pollinated and then tagged to record the number of days after pollination (DAP). All standard and conventional field practices (including fertilization, irrigation, and pest control) were followed during the growing season.

Moreover, samples were collected from watermelon fruit flesh at four developmental stages: 10, 18, 26, and 34 DAP. In general, during these key four fruit developmental stages, a watermelon’s phenotypic and metabolic changes are more prominent (Guo et al., 2015). Samples were immediately frozen in liquid nitrogen and stored at −80°C for further analysis. Three biological replicates were used for each of the mentioned analyses, i.e., measurement of flesh firmness, cell wall contents measurement, and RNA extraction for RT-qPCR. For the measurement of flesh firmness, all three fruits were cut into two halves, and flesh firmness was recorded from five different points.

### Measurement of Watermelon Flesh Firmness

Fruit flesh firmness was measured at different key developmental stages 10, 18, 26, and 34 days after pollination (DAP), using a penetrometer (GY-4, Zhejiang Top instrument Co., Ltd., Zhejiang, China) (Huang et al., 2016), equipped with 8 and 11 mm puncture probe, all fruits are cut into two parts in the longitudinal direction, and each fruit was measured from five different points; from the placental end (start), top (sun-side), bottom (earth-side), center region (heart of fruit), and the blossom end (base).

### Measurement of Cell Wall Contents in Watermelon Flesh

Cell wall material was extracted based on the methodology followed by Selvendran (1975). About 25 g of flesh sample were homogenized in 100 ml 80% ethanol using a homogenizer and water bath at 95°C for 20 min. The homogenized solution was centrifuged at 4000 rpm, 25°C, for 10 min. The pellet was collected on 20 μm NY20 nylon net filters and repeatedly washed with 80% ethanol, and finally, the acetone yielded colorless mass, which was oven-dried overnight at 35°C. The dried residue (alcohol-insoluble) is termed cell wall content. The cell wall fractions were then sequentially measured from cell wall content, and the pectin contents were measured according to the method followed by Jiménez et al. (2001).

### Preparation of RNA-Seq Libraries

RNA library preparation and sequencing were done as the method described by Wang et al. (2018). The extracted RNA samples were tested by a Nanodrop Nano Photometer (IMPLEN, GmbH, Munich, Germany). Purity (OD260 / 280), the concentration, and nucleic acid absorption peaks are detected by Agilent 2100 Bioanalyzer (Agilent Technologies, Santa Clara, CA, United States). Qubit2.0 was used for preliminary quantification, and Agilent 2100 was used to detect the library’s insert size. The q-PCR method is used for accurate quantification and effective concentration of libraries (effective library concentration >2 nM). After cluster generation, the library preparations were sequenced on an Illumina Hiseq 2500 platform and paired-end reads were generated. The raw sequencing data has been deposited to the SRA database NCBI under the accession numbers PRJNA682019.

### Quantification and Mapping to Watermelon Genome

TopHat v1.0.12 was used to align clean reads to the watermelon genome database^2^. Mapping of reading numbers to each gene was done by using HTSeq v0.6.1 (Anders et al., 2015), and then gene lengths and read counts mapped to the genes were used to calculate FPKM (Mortazavi et al., 2008).

### Differential Expression Analysis

The DESeq R package was used to conduct differential expression analysis of samples (Andino et al., 2016). The FDR was controlled by Benjamini Hochberg method to correct the *P-*values (Benjamini and Hochberg, 1995). Genes having expression Change ≥ 2, an adjusted *P*-value < 0.05, and FDR < 0.01 were identified as differentially expressed genes (Anders and Huber, 2010).

### KEGG Enrichment Analysis of DEGs and WGCNA for Identification of Correlated Gene-Networks

The statistical enrichment of DEGs in KEGG pathways was tested by KOBAS2.0 (Yi et al., 2011). WGCNA was performed in R (version 4.0.4) using default parameters to identify modules having highly correlated genes (Zhang and Horvath, 2005; Langfelder and Horvath, 2008). Based on normalized FPKM values, an adjacency matrix was constructed. The phenotype data was imported into the WGCNA package, and a correlation-based association between phenotype and gene-modules was conducted using the default settings. The adjacency matrix was further converted to a topological overlap matrix (TOM) within the WGCNA package. After building the networks, transcripts with similar expression patterns were classified into one module, and eigengenes (also called hub/key genes) for these modules were calculated. The hub genes of each module were exported using the default parameters for Cytoscape 3.8.2 (Shannon et al., 2003) export.

### Validation of Hub-Genes by Real-Time Quantitative PCR Analysis

The gene expression analysis using RT-qPCR of selected hub genes was carried out using three independent biological replicates for each sample (Kariyanna et al., 2020). Total RNA was isolated using a plant RNA purification kit (TIANGEN, Beijing, China) following the manufacturer’s protocol and treated with (RNase-free DNase) to remove residual genomic DNA. Complementary DNA (cDNA) was synthesized with reverse transcriptase following the manufacturer’s instructions (Takara, Tokyo, Japan). Primers were designed using the online tool Primer3 (v. 0.4.0), primer sequences were provided in Supplementary Table 1. RT-qPCR was carried out as protocol described previously (Jawad et al., 2020). Actin “*Cla016178*” was used as a reference gene (Kong et al., 2014).

### Phylogenetic Analysis of Key Genes

The protein sequences of candidate genes were downloaded from Watermelon Genome Database (see text footnote 2) and blast against *Cucumis sativus, Cucumis melo*, *Jatropha curcas*, *Ziziphus jujube*, *Populus trichocarpa*, and *Populus euphratica*. Phylogenetic trees were drawn by using MEGA 6 (version 10.1.8) software (Tamura et al., 2013), alignment of protein sequences was performed by ClustalW tool, and the neighbor-joining method with 1000 bootstrap replicates was used for the construction of tree (Li et al., 2012; Wei et al., 2014).

## Results

### Phenotypic Variation in Flesh Firmness During Different Fruit Developmental Stages

**FIGURE 1:**
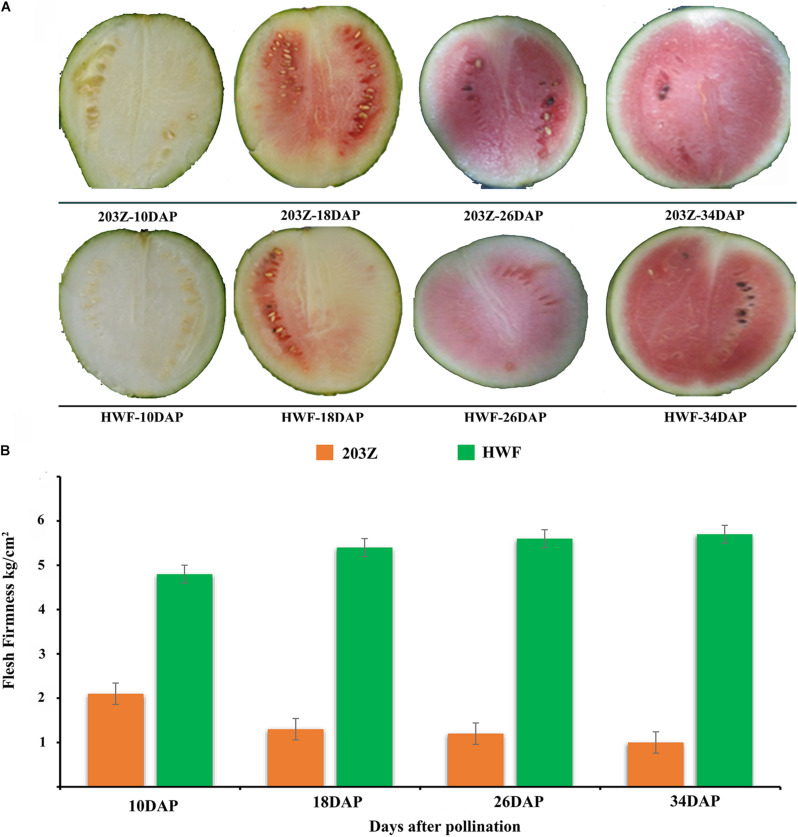
Phenotypic variation in flesh firmness during different fruit developmental stages (10, 18, 26, and 34 DAP). **(A)** 203Z and HWF watermelon fruits at 10, 18, 26, and 34 DAP, **(B)** Changed in flesh firmness of 203Z and HWF at 10, 18, 26, and 34 DAP.

Flesh firmness was measured at all key development stages: 10, 18, 26, and 34 DAP, and significant variations in flesh firmness were observed between HWF and 203Z. Flesh firmness of HWF increased with the developmental stages; the minimum flesh firmness was measured (4.8 kg/cm^2^) at 10 DAP, and the maximum was measured (5.7 kg/cm^2^) at 34 DAP. In contrast, the flesh firmness of 203Z decreased with the developmental stages; the maximum firmness was measured (2.1 kg/cm^2^) at 10 DAP, and the minimum was measured (1.00 kg/cm^2^) at 34 DAP. Altogether, the flesh firmness of HWF was significantly higher than the flesh firmness of 203Z (Figure 1).

### Changes in the Content of Cell Wall Components During Key Fruit Developmental Stages

**FIGURE 2:**
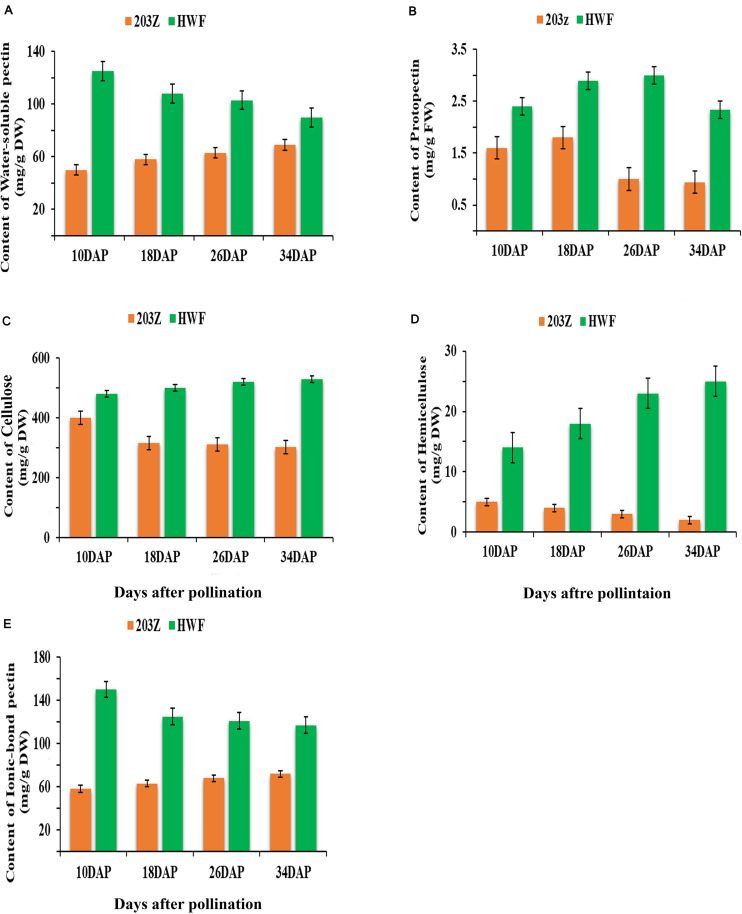
Changes in cell wall components’ content during fruit development stages (10, 18, 26, and 34 DAP) in 203Z and HWF, green color represents HWF, and orange color represents 203Z. **(A)** Content of water-soluble pectin (mg/g DW), **(B)** Content of protopectin (mg/g FW), **(C)** Content of cellulose (mg/g DW), **(D)** Content of hemicellulose (mg/g DW), **(E)** Content of ionic-bond pectin (mg/g DW).

Cell-wall contents were measured at four key development stages: 10, 18, 26, and 34 DAP. The variation in cell wall contents can be easily visualized in Figure 2 during fruit developments; the protopectin content shows variation in two test materials. Content in HWF firstly increased and then started to decrease after 26 DAP, while 203Z content starts to decrease after 18 DAP until maturity. Water-soluble pectin shows significant differences between the two materials, the content in HWF is significantly higher than 203Z, and the trend in HWF decreased. In contrast to 203Z, the content increased with developmental stages. Like water-soluble pectin, ionic-bond pectin content also showed significant differences between HWF and 203Z and followed a similar trend. The content of cellulose and hemicellulose shows an increased-decreased trend, and in HWF and 203Z, the trend of cellulose content in HWF and 203Z is opposite, increased in HWF with developmental stages while in 203Z decreased from the beginning 10 DAP, and there is no significant variation observed between HWF and 203Z during four developmental stages. The hemicellulose content trend is similar to cellulose, but hemicellulose content between HWF and 203Z showed significant differences; the content in HWF was measured 2-fold higher than in 203Z. The combined observation of cell-wall contents showed that the percentage cell wall contents in HWF are significantly higher than the percentage cell-wall contents in 203Z and the content of cell-wall components shows significant variation between two test materials with developmental stages.

### Genome-Wide Transcriptome Analyses of 203Z and HWF

High-throughput RNA sequencing (RNA-Seq) generated a total of 199.11 Gb clean reads from 24 samples collected at four key developmental stages: 10, 18, 26, and 34 DAP, from two materials HWF and 203Z. The Clean Data of each sample reached 6.76 Gb, and the Q30 base percentage was 91.32% and above (Supplementary Table 2). The clean reads of each sample were compared with the designated watermelon reference genome (see text footnote 2), and the alignment efficiency varied from 81.82 to 86.33%. We carried out the predictive analysis of alternative splicing, optimization analysis of gene structure, and discovering new genes based on the comparison results. We discovered 902 new genes, of which 587 were functionally annotated. Based on the comparison results, gene expression analysis was performed. Differentially expressed genes were selected according to their expression in different samples and performed functional annotation and enrichment analysis.

A total of 7965 differentially expressed genes (DEGs) were identified between HWF and 203Z. For the investigation of genes related to flesh firmness, we compared and analyzed DEG’s at four key developmental stages between two parental lines; 10 days after pollination (203Z-10DAP vs. HWF-10 DAP), 18 days after pollination (203Z-18DAP vs. HWF-18DAP), 26 days after pollination (203Z-26DAP vs. HWF-26DAP), and 34 days after pollination (203Z-34DAP vs. HWF-34DAP), there were 886, 981, 429, and 283 DEGs were expressed at these key developmental stages, respectively (Supplementary Figure 1). Common and unique genes between the four key developmental stages were illustrated in the Venn diagram (Supplementary Figure 2). In total, 1432 genes were significantly up-regulated, and 1147 genes were significantly down-regulated in HWF compared with 203Z at four key developmental stages (Table 1).

**TABLE 1:** Pairwise comparison of up and down-regulated DEGs between 203Z VS HWF at 10, 18, 26, and 34 DAP.

Groups	Total DEGs	Up regulated	Down regulated
203Z-10-DAP_vs_HWF-10-DAP	886	351	535
203Z-10-DAP_vs_203Z-118-DAP	3,484	1,276	2,208
203Z-l0-DAP_vs_203Z-26-DAP	4,735	1,858	2,877
203Z-10-DAP_vs_203Z-34-DAP	3,472	1,351	2,121
HWF-10-DAP_vs_HWF-18-DAP	1,725	802	923
HWF-10-DAP_vs_HWF-26-DAP	4,243	1,868	2,375
HWF-10-DAP_vs_HWF-34-DAP	4,745	2,069	2,676
203Z-l8-DAP_vs_HWF-l8-DAP	981	665	316
203Z-18-DAP_vs_203Z-26-DAP	1,516	781	735
203Z-18-DAP_vs_203Z-34-DAP	820	411	409
HWF-18-DAP_vs_HWF-26-DAP	2,882	1,288	1,594
HWF-18-DAP_vs_HWF-34-DAP	3,437	1,483	1,954
203Z-26-DAP_vs_HWF-26-DAP	429	217	212
203Z-26-DAP_vs_203Z-34-DAP	218	78	140
HWF-26-DAP_vs_HWF-34-DAP	621	329	292
203Z-34-DAP_vs_HWF-34-DAP	283	199	84

Gene ontology (GO) enrichment analysis of the DEGs was performed to understand gene functions associated with watermelon’s flesh firmness. For both up- and down-regulated genes, the most three significantly enriched GO terms in the “biological process”, “cellular component”, and “molecular function” groups were “single-organism process”, “cellular process”, and “metabolic process” in the biological process category; “cell part”, “cell”, and “organelles” in the cellular components category; and “binding activity”, “catalytic activity”, and “transporter activity” in the molecular function category.

**FIGURE 3:**
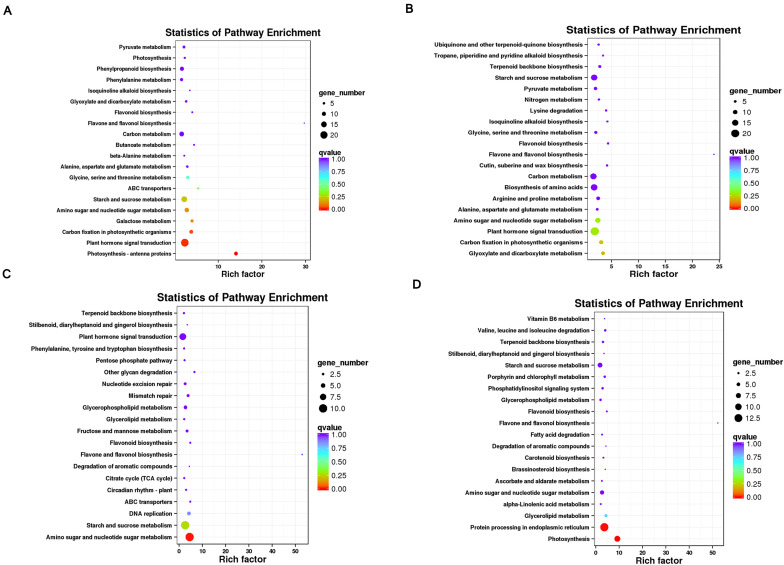
KEGG enrichment analysis of DEGs found by pairwise comparisons among different developmental stages of 203Z and HWF. **(A)** 203Z vs. HWF at 10 DAP, **(B)** 203Z vs. HWF at 18 DAP, **(C)** 203Z vs. HWF at 26 DAP, **(D)** 203Z vs. HWF at 34 DAP.

To make out DEGs functions, we also conducted a Kyoto Encyclopedia of Genes and Genomes (KEGG) enrichment analysis. The 1432 up-regulated and 1147 down-regulated genes were enriched in 112 metabolic pathways, including five large categories: Cellular processes, Environmental information processing, Genetics information process, Metabolism, and Organic systems (Organismal systems). Among the categories, the secondary metabolic pathway with the largest number of DEGs is plant hormone signal transduction. In the environmental information process category, followed by carbon metabolism and starch and sucrose metabolism, both pathways are in the metabolic classification category. In this study, the category metabolism contains the most KEGG pathways, indicating that differentially expressed genes involved in metabolic pathways may contribute to the variation of flesh firmness in watermelon (Figure 3).

### WGCNA for Identification of Highly Connected “Hubs” and Correlated Gene-Modules

**FIGURE 4:**
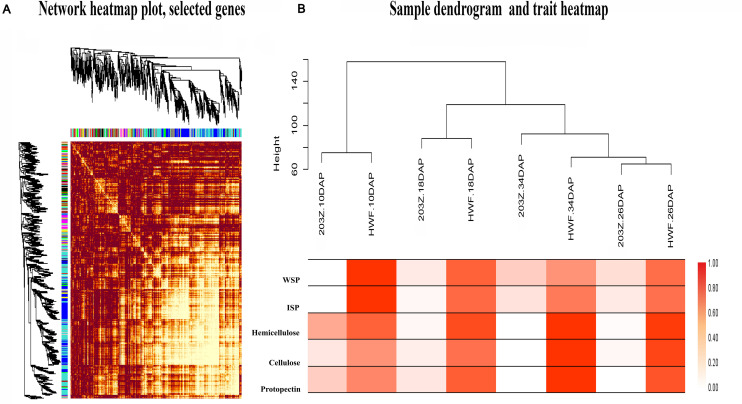
Network heatmap plot of selected genes, sample dendrogram, and trait heatmap. **(A)** Hierarchical clustering of the topological overlap matrix (TOM) of the differentially expressed genes. **(B)** Sample dendrogram and trait heatmap, to detect the outliers. The height of the scale is the distance matrix between the clusters.

Weighted genes co-expression network analysis is an omics analysis tool, defining the networks that continuously link all variables and then cluster the most highly co-expressed variables inflexibly defined modules. Herein, we constructed the transcriptome dataset of two materials, HWF and 203Z, at four key developmental stages, i.e., 10, 18, 26, and 34 DAP. All genes expressed during these stages between two materials were entered into WGCNA with FPKM values to reveal the highly correlated genes. An adjacency matrix was generated among the two materials at four stages by using DEGs resulting in 67 distinct gene-modules. Each module is referred to the distinct groups formed by the clustering of genes, and an arbitrary color has designated each module to distinguish between them, presented as a clustergram and network heatmap (Figure 4A). Water-soluble pectin (WSP), ionic bound pectin (ISP), cellulose, hemicellulose, and protopectin were used as phenotypic data for each development stage analysis of module trait correlation. To identify the sample outliers and sample trait correlation, we constructed the sample dendrogram and trait heatmap (Figure 4B). Eight samples are clustered into two main clades, with one clade then further divided into sub-clades, and each clade corresponded to a different trait. As expected, there were no outliers identified from sample sets, which increased the confidence on sample trait correlation. Afterward, eigengene-trait correlation analysis was performed for the relationships between module eigengenes and phenotypic traits (cell wall contents), showing that the correlation coefficients varied widely. The results showed that there were different modules significantly (*r*^2^> 0.7, *P* < 0.05) correlated with phenotypic traits (Supplementary Figure 3).

### Module Analysis Based on WGCNA

**FIGURE 5:**
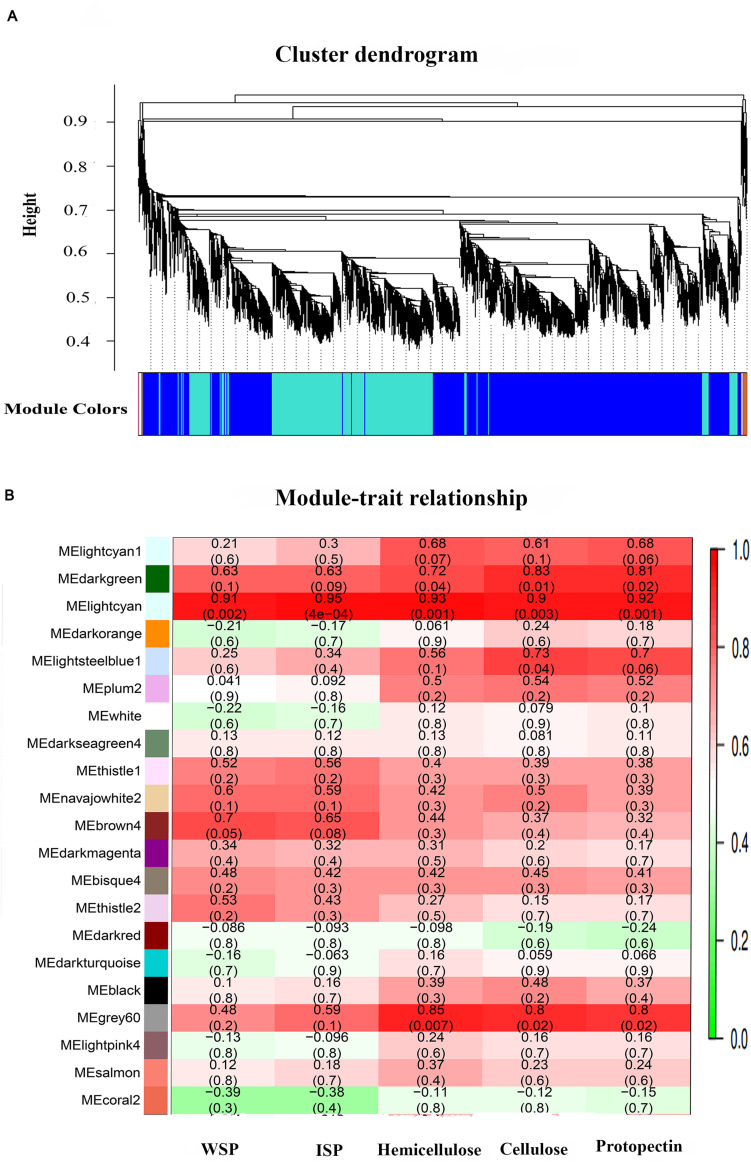
Gene cluster dendrogram based on co-expression network analysis in 203Z and HWF and module trait relationship. **(A)** Hierarchical clustering presenting 67 modules having co-expressed genes. Each leaflet in the tree corresponds to an individual gene. **(B)** Module-trait associations based on Pearson correlations. Color key from green to red represents r^2^ values from –1 to 1.

Among 67 gene-modules, only three modules showed a significant correlation with phenotypic traits. MEdarkgreen module with 101 genes was strongly correlated with cellulose (*r*^2^ = 0.83, *p*-value = 0.01) and protopectin (*r*^2^ = 0.81, *p*-value = 0.02). MElightcyan module with total 137 genes showed a significant correlation with cellulose (*r*^2^ = 0.9, *p*-value = 0.003), water-soluble pectin (*r*^2^ = 0.91, *p*-value = 0.002), and protopectin (*r*^2^ = 0.92, *p*-value = 0.001), and MEgrey60 module consisting of 115 genes showed a significant correlation with cellulose (*r*^2^ = 0.8, *p*-value = 0.02), hemicellulose (*r*^2^ = 0.85, *p*-value = 0.007), and protopectin with (*r*^2^ = 0.8, *p*-value = 0.02). The detailed description of gene-modules and traits correlations is presented in Figures 5A,B.

All the genes from each of these three modules were selected based on intramodular gene significance, and their edges and nodes were calculated within the WGCNA R package (Supplementary Table 3). This information was exported to Cytoscape for gene-networks visualizations. Top genes from all three significantly correlated modules were extracted and annotated in the watermelon annotation database. Finally, two genes *Cla012351* (Cellulose synthase) and *Cla004251* (Pectinesterase) from MEdarkgreen module, three genes Cla*004120* (ERF), *Cla009966* (Cellulose synthase), and *Cla006648* (Galactosyltransferase) from MElightcyan module, and three genes *Cla007092* (ERF), *Cla004119* (Glycosyltransferase), and *Cla018816* (Xyloglucan endotransglucosylase/hydrolase) from MEgrey60 module, are identified as hub-genes. These putative candidate genes are highlighted in red in the gene-networks; the clear features of these gene-networks can easily observe in Cytoscape display (Supplementary Figure 4).

### Phylogenetic Analysis of Candidate Genes

Different distinct clusters were formed as a result of phylogenetic analysis. Watermelon pectinesterase gene *Cla004251* had high homology with pectinesterase/pectinesterase inhibiter of *Cucumis sativus* and followed by pectinesterase/pectinesterase inhibiter 34 of *Jatropha curcas*, *Ziziphus jujube*, *Populus trichocarpa*, and *Populous euphratica*. Similarly, xyloglucan glycosyltransferase gene *Cla006648* shows homology with xyloglucan glycosyltransferase GT14 of *Cucumis sativus* and xyloglucan glycosyltransferase of *Cucumis melo*, and another xyloglucan glycosyltransferase gene *Cla018816* shows high homology with xyloglucan glycosyltransferase/hydrolase protein 23-like *Cucumis sativus* and xyloglucan glycosyltransferase/hydrolase of *Cucumis melo*. Glycosyltransferase genes *Cla004119* had high homology with Glycosyltransferase *At5g03795* of *Cucumis sativus* and Glycosyltransferase *At5g03795* of *Cucumis melo*. Cellulose synthase gene *Cla012351* had high homology with cellulose synthase, a catalytic subunit of *Cucumis sativus* and *Cucumis melo*, and another cellulose synthase gene *Cla009966* had homology with xyloglucan glycosyltransferase 12 *Cucumis melo* and xyloglucan glycosyltransferase *Cucumis sativus*, and the ethylene-responsive element genes *Cla007092*, *Cla004120* had high homology with *ERF113* of *Cucumis sativus* and *ERF* of *Cucumis melo* (Supplementary Figure 5).

### RT-qPCR to Verify the Accuracy of Transcriptome Data

**FIGURE 6:**
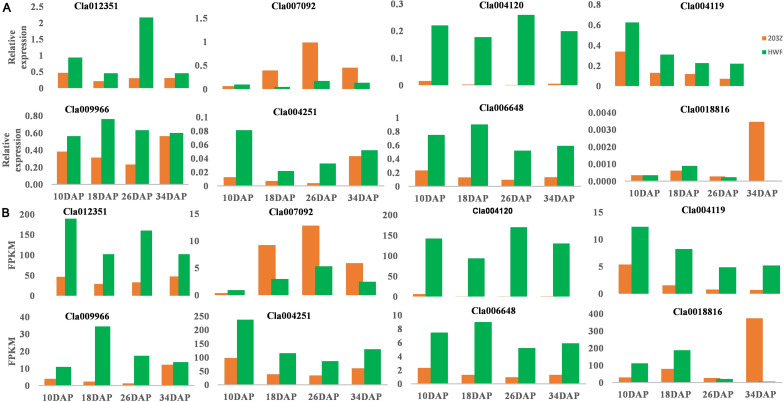
Relative expressions of selected genes by RT-qPCR to verify the accuracy of FPKM values by RNA-sequencing. **(A)** Relative expressions of selected genes by RT-qPCR. **(B)** FPKM values by RNA-Sequencing. *X*-axis represents the key four fruit development stages, 10, 18, 26, and 34 DAP. *Y*-axis represents the gene relative expression levels by RT-qPCR and FPKM values by RNA sequencing, green color represents HWF, and orange color represents 203Z.

The accuracy of the transcriptome sequencing data is the prerequisite for identifying differentially expressed genes and subsequent enrichment analysis of GO and KEGG functions. To verify the reliability of transcriptome sequencing data, we selected eight hub genes to check the relative expression in 203Z and its near-isogenic line HWF. The eight genes selected include pectinesterase gene (*Cla004251*), xyloglucan endoglycosyl transferase genes (*Cla006648* and *Cla018816*), glycosyltransferase gene (*Cla004119*), cellulose synthase gene (*Cla012351* and *Cla009966*), and ethylene response element genes (*Cla007092* and *Cla004120*). RT-qPCR analysis revealed that the trend of gene expression by transcription measured (FPKM) and relative expression by the RT-qPCR method was consistent (Figure 6). The results indicate the validation of transcriptome data and gene expressions in soft and hard materials were different, thus validating those key selected genes were the true candidates involved in fruit flesh firmness of watermelon.

## Discussion

Fleshy fruits development and ripening are complex biological processes that occupied a significant stage in horticulture; the trait is regulated by various environmental, hormonal, and gene regulation factors (Wechter et al., 2008). Herein, we applied weighted gene co-expression network analysis (WGCNA) to manipulate a multivariate transcriptome dataset sampled from different developmental stages of two materials, HWF and 203Z. By using WGCNA, we find significant modules associated with the flesh firmness trait of watermelon.

### Effect of Cell Wall Contents on the Flesh Firmness of Watermelon at Different Developmental Stages

Variation in flesh firmness of fruits is often attributed to the changes in the strength of the primary cell wall and intercellular connections (Harker et al., 1997). Cell wall metabolism is an important change related to flesh firmness during fruit development and ripening (Sun et al., 2012). The reduction in the firmness of fruit during development involves the coordinated series of modification to the polysaccharide components of the primary cell wall and middle lamella (Brummell, 2006). In the cell wall of fruit, the main components included pectin, cellulose, and hemicellulose, which form an interwoven network structure with different types of cell wall modifying proteins. The initial cause of fruit ripening and change in flesh firmness is the destruction of the cell wall internal structure, which is mainly driven by the degradation of the pectin substance in the flesh cells, which caused the separations of flesh cells from each other. During fruit ripening, different types of cell wall metabolism-related enzymes work together to cause pectin polymers’ degradation (Goulao and Oliveira, 2008), resulting in decreased cell-to-cell tightness and fruit softening. Pectin degradation is a common phenomenon existing in fruit ripening and change in flesh firmness of fruits, which is mainly manifested by the increase in water-soluble pectin content.

In this study, the soft flesh types watermelon 203Z, the water-soluble pectin content of the fruit flesh cells increased with the decreased in flesh firmness, while in the hard flesh type watermelon HWF. The water-soluble pectin content of the fruit flesh cells decreased with the increase of flesh firmness, which were related to the firmness of fruit flesh. The opposite trend of water-soluble pectin with flesh firmness during key developmental stages showed that the firmness change of watermelon flesh has a strong correlation with water-soluble pectin content. Many related studies have shown that during the development and storage of fruits, the decrease in the flesh firmness is often accompanied by the presence of water-soluble pectin (Fils-Lycaon and Buret, 1990; Wei et al., 2010; Gwanpua et al., 2014; Sun et al., 2020), our results are in agreement with previous studies. Ionic-bound pectin is another major type of pectin. The soluble polysaccharide polymer is mainly the combination of lysed cell walls. An increase in the content of ionic-bond pectin indicates that the calcium bridge is damaged and the flesh firmness is decreased. Related studies had shown that before the fruit maturity, the content of ionic-bond pectin in the fruit increase continuously, and then the fruit matures and softens. The increase in the actual ionic-bound pectin content may be the direct cause of the decrease in fruit flesh firmness (Xue et al., 2006; Draye and Van Cutsem, 2008). As the watermelon fruit develops and mature, the content of ionic-bound pectin shows variation in two test materials in contrast to flesh firmness, i.e., the content in 203Z (soft material) increased with the decrease of flesh firmness, and the trend in HWF (hard material) is opposite. The realization of variation in ionic-bond content is mainly due to two test materials’ different flesh firmness trends during development. Many studies showed that the content of covalently bound pectin is consistent with the change in flesh firmness, that is, decrease with the decrease of fruit firmness, in grapes after ripening covalently bound pectin disaggregated into water-soluble loose pectin and ion-bonded pectin (Deng et al., 2016) studied on pear fruit with different fruit textures found that during the fruit development, the covalently bound pectin content increased, while during storage, the pears with higher firmness had higher covalently bound pectin content (Wei et al., 2015). Protopectin also showed an increase (decrease) trend in 203Z and HWF, but the variation in content is not significant. Cellulose and hemicellulose are the main components of the cell wall, providing mechanical strength to cell-wall structure (Burton et al., 2010; Guo et al., 2015). Studies suggested that during the ripening process of strawberry fruits, the cellulose content decreases continuously, which is an important factor of the transformation (Xue et al., 2006; Zhao, 2007).

In contrast, there is no role of hemicellulose content during the change in firmness. Softening of strawberry fruit during development is related to the dissolution and depolymerization of the pectin, not hemicellulose degradation (Koh and Melton, 2002; Rosli et al., 2004). In this study, cellulose and hemicellulose content in fruit flesh of 203Z and HWF were consistent with the changing trend of flesh firmness. Based on the different appearance of cell-wall contents in two materials during the development of watermelon fruit, it can be assumed that these components are playing a vital role in the variation of watermelon fruit flesh firmness.

### Candidate Genes in Different Modules Correlated With the Flesh Firmness of Watermelon

The genes related to metabolism that promotes changes in the composition of cell wall metabolites may also be in regulating the flesh firmness of watermelon fruits; these genes mainly include pectin methylesterase (*PME*), polygalacturonase (*PG*), cellulose synthase, and alpha-galactosidase genes. The cellulose synthase family genes are reported in *Arabidopsis thaliana* and the possible involvement in cellulose deposition in cell-skeleton (Richmond and Somerville, 2000; Paredez et al., 2006; Suzuki et al., 2006). In this study, seven cellulose synthase genes are expressed at different development stages in transcriptome data sets, among which one gene *Cla012351* was identified as the hub/candidate gene in darkgreen module by WGCNA; the gene expressed differentially in two test materials, the expression in HWF (hard flesh) are significantly higher than the 203Z (soft flesh), and based on WGCNA analysis and expression in test materials, we can assume that the *Cla012351* (Cellulose synthase) gene might be involved in the construction of cell wall skeleton of watermelon fruit flesh cells during fruit development. Xyloglucan endo-transglycosylase/hydrolase (*XTH*) family genes are mainly involved in plant development and fruit maturation; xyloglucan is the main hemicellulose component in the primary cell wall of dicotyledonous plant cells. It becomes an important structural substance in the cell wall (Muñoz-Bertomeu et al., 2013). A decrease in fruit firmness during maturation is caused by rapid hydrolysis of pectin, cellulose, and Xyloglucan (Redgwell et al., 1997; Miedes and Lorences, 2009). In this study, 21 genes of the XTH family showed different expressions in two test materials; among them, *Cla018816* (Xyloglucan endotransglucosylase/hydrolase) and *Cla006648* (Galactosyltransferase), are identified as candidate/hub genes. Studies suggested that XTH family genes possible involvement in the construction and disassembly of cell wall architecture (Matsui et al., 2005). The enzymes that played roles in the disintegration of pectin in fruit cell-wall are pectinesterase and polygalacturonase. Pectinesterase act as a catalyst for the hydrolytic de-esterification of pectin, which causes the pectic chain esterification, which is further hydrolyzed to pectate by polygalacturonase, causing loss in fruit firmness during fruit ripening, and in this study 30 differentially expressed genes were observed in transcriptome data, by WGCNA we identified *Cla004251* (Pectinesterase) gene. In transcriptome data, we observed expression in HWF (hard-flesh) is significantly higher than the expression in 203Z (soft-flesh) watermelon fruits. Studies showed, the degradation of pectin during fruit developments is catalyzed by cell wall enzyme pectin methylesterase (PME). The expression of PME is influenced by ethylene in fruits (Willats et al., 2001); the activity of pectinesterases can be regulated by pectinesterase inhibitors (Jolie et al., 2010; Pinzón-Latorre and Deyholos, 2013), and in this study the phylogenetic analysis of *Cla004251* gene showed high homology with the pectinesterase/pectinesterase inhibiter of *Cucumis sativus*, *Jatropha curcas, Ziziphus jujube, Populus trichocarpa*, and *Populous euphratica.* From these results we can assume the possible involvement of *Cla004251* to strengthening and solubilization of pectin during watermelon fruit development, which in turn is related to changes in fruit flesh firmness.

Fleshy fruits can be classified into two classes: climacteric fruits and non-climacteric fruits (McMurchie et al., 1972). Climacteric fruits have a sharply increased ethylene concentration during maturation, while non-climacteric fruits have low ethylene synthesis at maturity. During the fruit ripening, ethylene biosynthesis and signal transduction are regulated by upstream transcription factors, such as *AP2 / ERF* (Chung et al., 2010). These factors regulate the development and maturation of fruits positively or negatively: the multiple ERF transcription factors have been found in tomatoes that regulate fruit maturation and softening of fruit (Zhang et al., 2009; Lee et al., 2012; Liu et al., 2014). The regulation of fruit maturity and changes in flesh firmness of watermelon fruits during development by ethylene cannot be ignored. Strawberries are considered as investigative model crop of non-climacteric fruits. The treatment of strawberry with exogenous ethylene at the initial ripening period can accelerate fruit color change and change in fruit firmness (Given et al., 1988; Iannetta et al., 2006). In this study by comparative transcriptome analysis, we identified 26 genes related to ethylene biosynthesis and signaling pathways; the genes include ACC synthase (*ACS*), ACC oxidase (*ACO*), ethylene receptor (*ETR*), and ethylene-responsive factor (*ERF*) genes by correlated network analysis (WGCNA). We found two *ERF* genes, i.e., *Cla004120* (Ethylene-responsive transcription factor 1) and *Cla007092* (Ethylene responsive transcription factor 2a), as candidate/hub genes and observed the significant differences in expression in two test materials. The two genes showed opposite trend of expression; *Cla007092* gene expression level is high in soft material 203Z as in hard flesh HWF, while the expression of *Cla004120* is significantly higher in hard material than in soft flesh watermelon. Studies in tomato suggested that ethylene regulates fruit genes, including fruit-specific polygalacturonase (PG), which is involved in depolymerization of cell wall pectin during ripening, pectin methylesterase (*PME*), which provides accessibility to pectin by PG (Cara and Giovannoni, 2008). Based on this study, we speculate that ethylene-responsive elements regulate the cell wall biosynthesis genes involve in flesh firmness change in watermelon fruit. But the mechanism of interaction is unclear, which provides important clues for our further study of the molecular mechanism of watermelon flesh firmness variation.

## Data Availability Statement

The datasets presented in this study can be found in online repositories. The names of the repository/repositories and accession number(s) can be found below: NCBI BioProject: PRJNA682019.

## Author Contributions

LG, SZ, and WL conceived the research and designed the experiments. MA and LG designed the experiments. MA and MU data analysis and wrote the whole manuscript. SZ and LG are involved in sample collection and RNA extraction work. XL provided valuable experimental materials and methods. MK, CG, and PY assisted the laboratory work and facilitate materials. HZ checked the manuscript. All authors read and approved the final manuscript.

## Conflict of Interest

The authors declare that the research was conducted in the absence of any commercial or financial relationships that could be construed as a potential conflict of interest.

## Footnotes

1 http://www.fao.org/faostat

2 http://cucurbitgenomics.org/organism/1

## Supplementary Material

Supplementary Figure 1 Volcano diagram of differentially expressed genes between 203Z and HWF at 10 DAP **(A)**, 18 DAP **(B)**, 26 DAP **(C)**, and 34 DAP **(D)**. *Y*-axis represents value A (log2 transformed mean expression level). *X*-axis represents value M (log2 transformed fold change), red dots represent up regulated DEGs, green dots represent down regulated DEGs. Black dots represent non-DEGs.

Supplementary Figure 2 Venn diagram showing common and unique genes between two HWF and 203Z at four key developmental stages; A1-VS-B1 (203Z10DAP vs. HWF10DAP), A2-VS-B2 (203Z18DAP vs. HWF18DAP), A3-VS-B3 (203Z26DAP vs. HWF26DAP), and A4-VS-B4 (203Z34DAP vs. HWF34DAP).

Supplementary Figure 3 Module-trait associations based on Pearson correlations. Color key from green to red represents r^2^ values from −1 to 1.

Supplementary Figure 4 Network visualization of hub genes **(A)** Dark-green module gene, **(B)** light-cyan **(C)** grey60 module gene. The red color heightened cells indicate the hub-genes.

Supplementary Figure 5 Phylogeny of watermelon genes involved in regulation and transport of flesh firmness. The phylogenetic tree was created from protein sequences by maximum likelihood method.

Supplementary Table 1 Primer used for q-RT-PCR.

Supplementary Table 2 Comprehensive Evaluation of RNA-Seq Data: A: 203Z, B: HWF. A Total Mapped Reads (%) = Unique Match (%) + Multi-position Match (%), are the percentages of clean reads align to reference genome. b Q30 (%) are the percentages of reads with Phred qualities scores over than 30.

Supplementary Table 3 Cytoscape Input values of three gene modules.

Supplementary Table 4 Identified QTLS for fruit flesh firmness in different crops.
