# Supplementary figures and images for: Identification of Key Gene Networks Associated With Cell Wall Components Leading to Flesh Firmness in Watermelon
Source: Front Plant Sci. 2021 Jun 22;12:630243. doi: 10.3389/fpls.2021.630243 (PMC8259604; doi:10.3389/fpls.2021.630243)

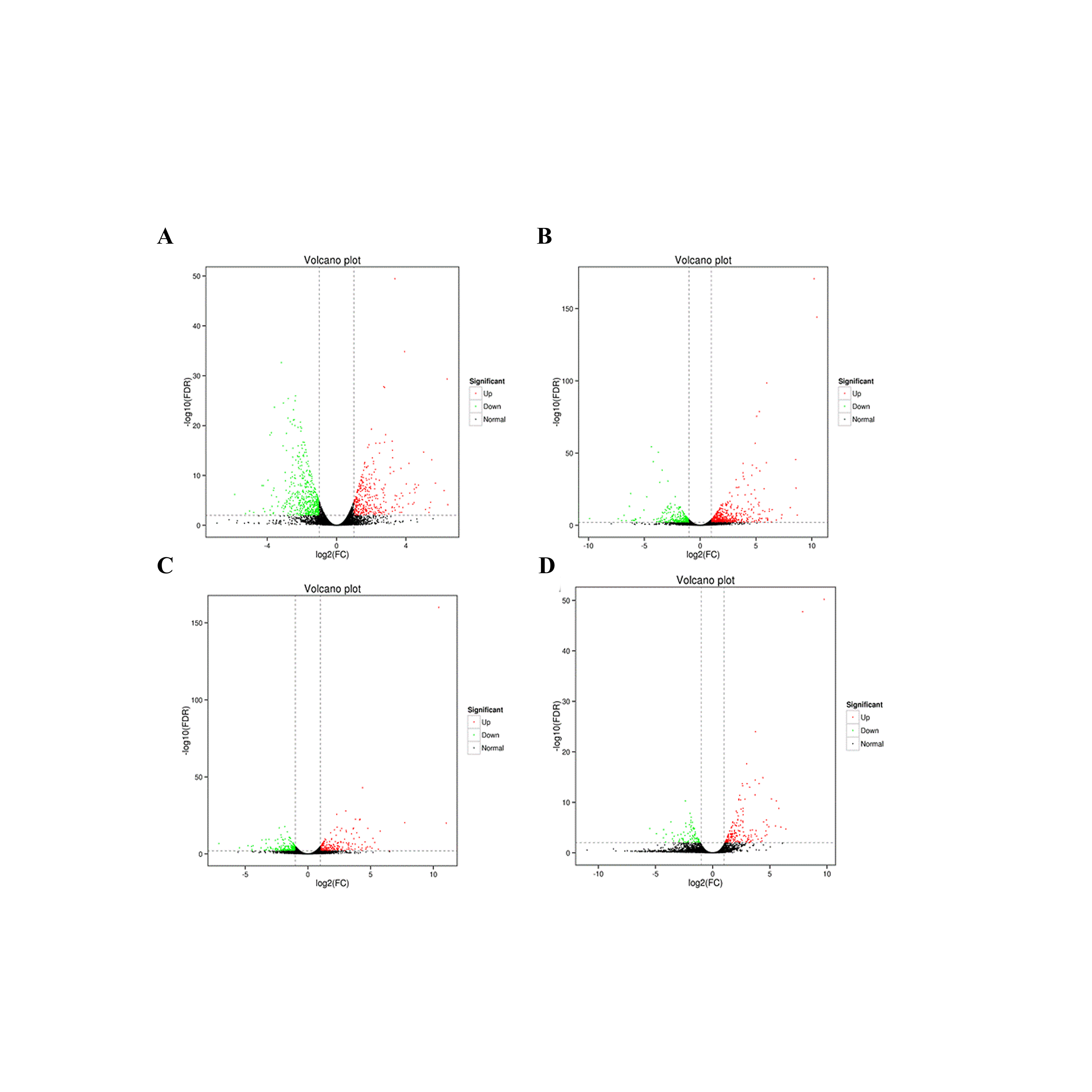

Supplement: Supplementary Figure 1 — Volcano diagram of differentially expressed genes between 203Z and HWF at 10 DAP (A), 18 DAP (B), 26 DAP (C), and 34 DAP (D). Y-axis represents value A (log2 transformed mean expression level). X-axis represents value M (log2 transformed fold change), red dots represent up regulated DEGs, green dots represent down regulated DEGs. Black dots represent non-DEGs. [file Image_1.TIF]

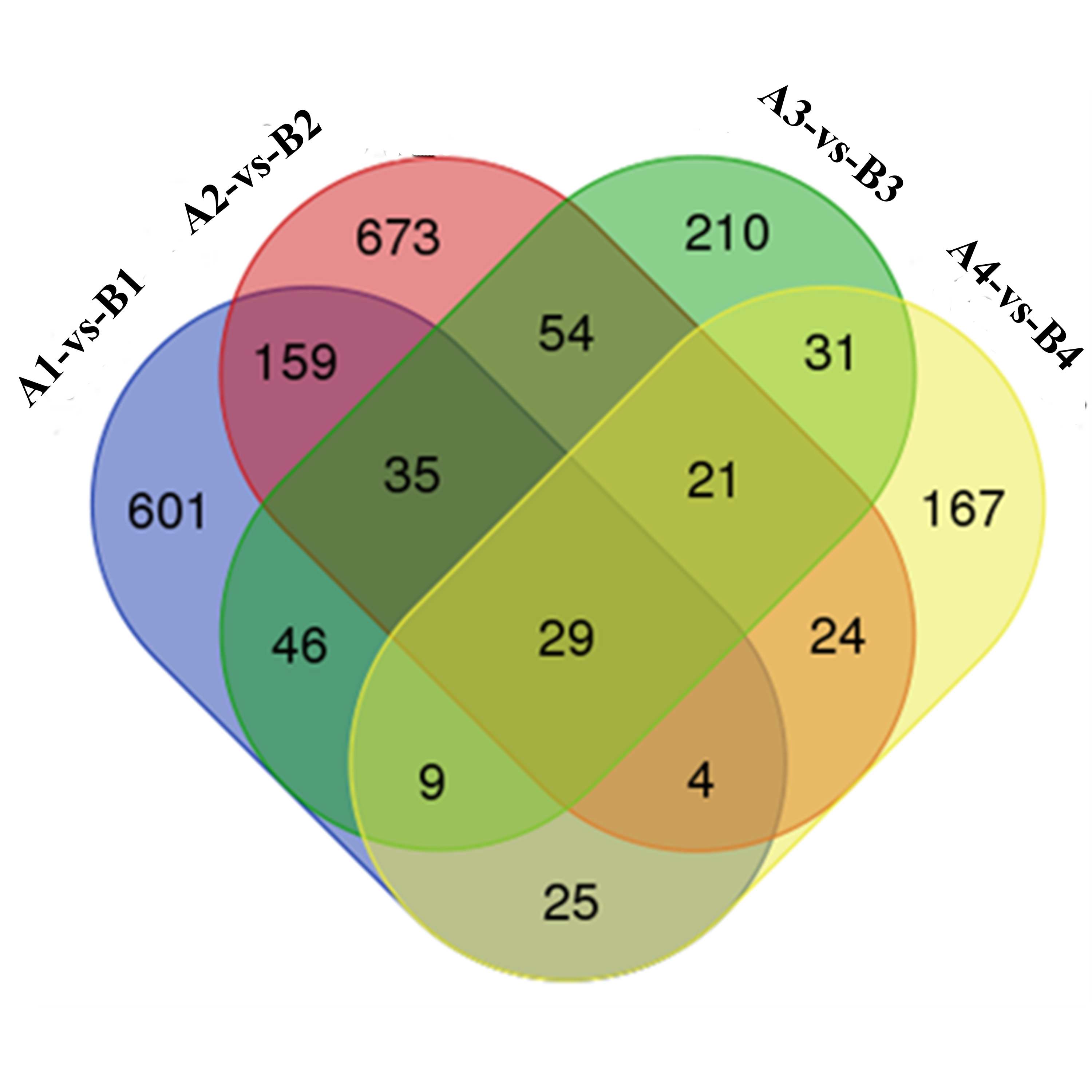

Supplement: Supplementary Figure 2 — Venn diagram showing common and unique genes between two HWF and 203Z at four key developmental stages; A1-VS-B1 (203Z10DAP vs. HWF10DAP), A2-VS-B2 (203Z18DAP vs. HWF18DAP), A3-VS-B3 (203Z26DAP vs. HWF26DAP), and A4-VS-B4 (203Z34DAP vs. HWF34DAP). [file Image_2.TIF]

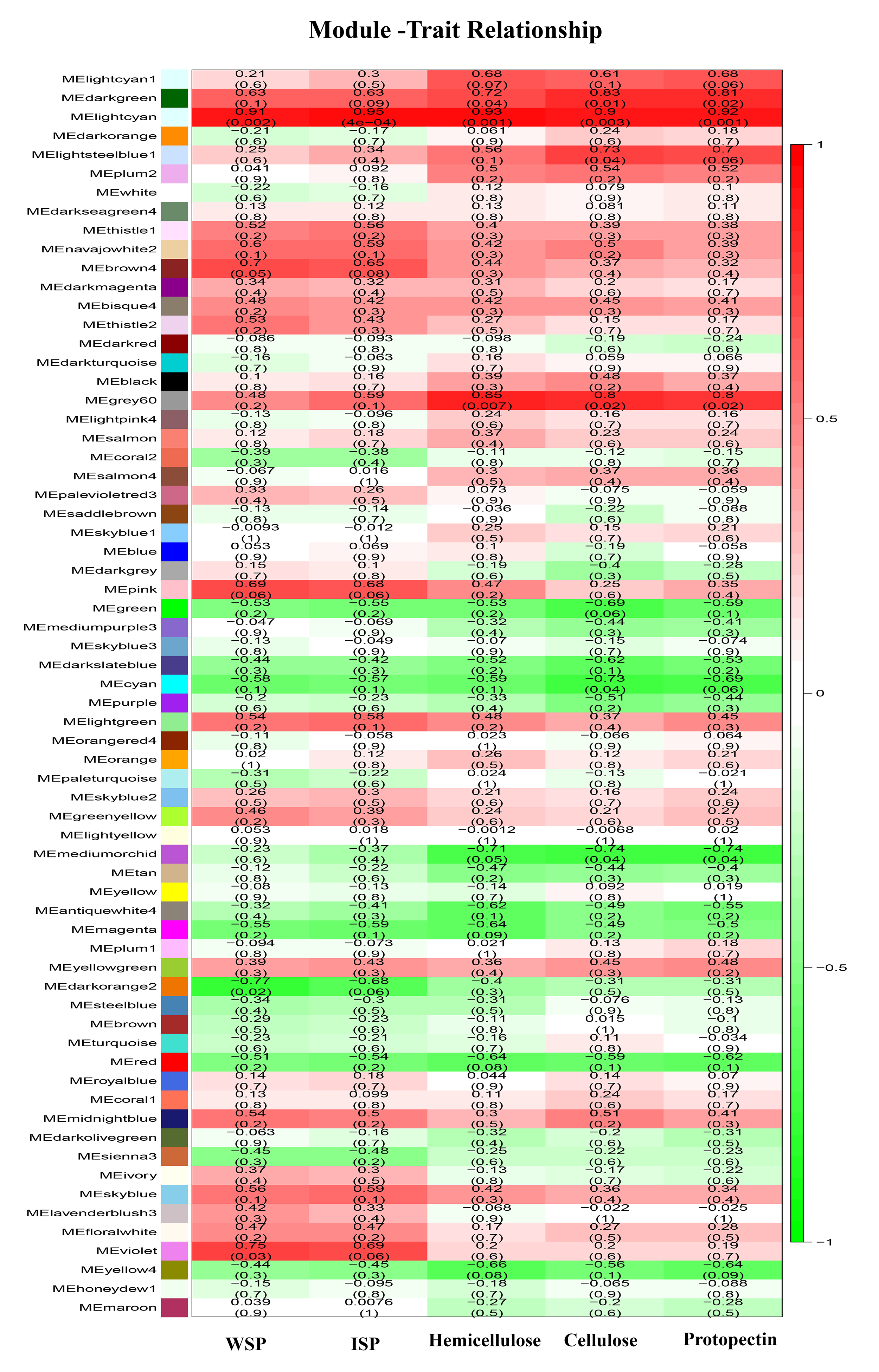

Supplement: Supplementary Figure 3 — Module-trait associations based on Pearson correlations. Color key from green to red represents r2 values from −1 to 1. [file Image_3.TIF]

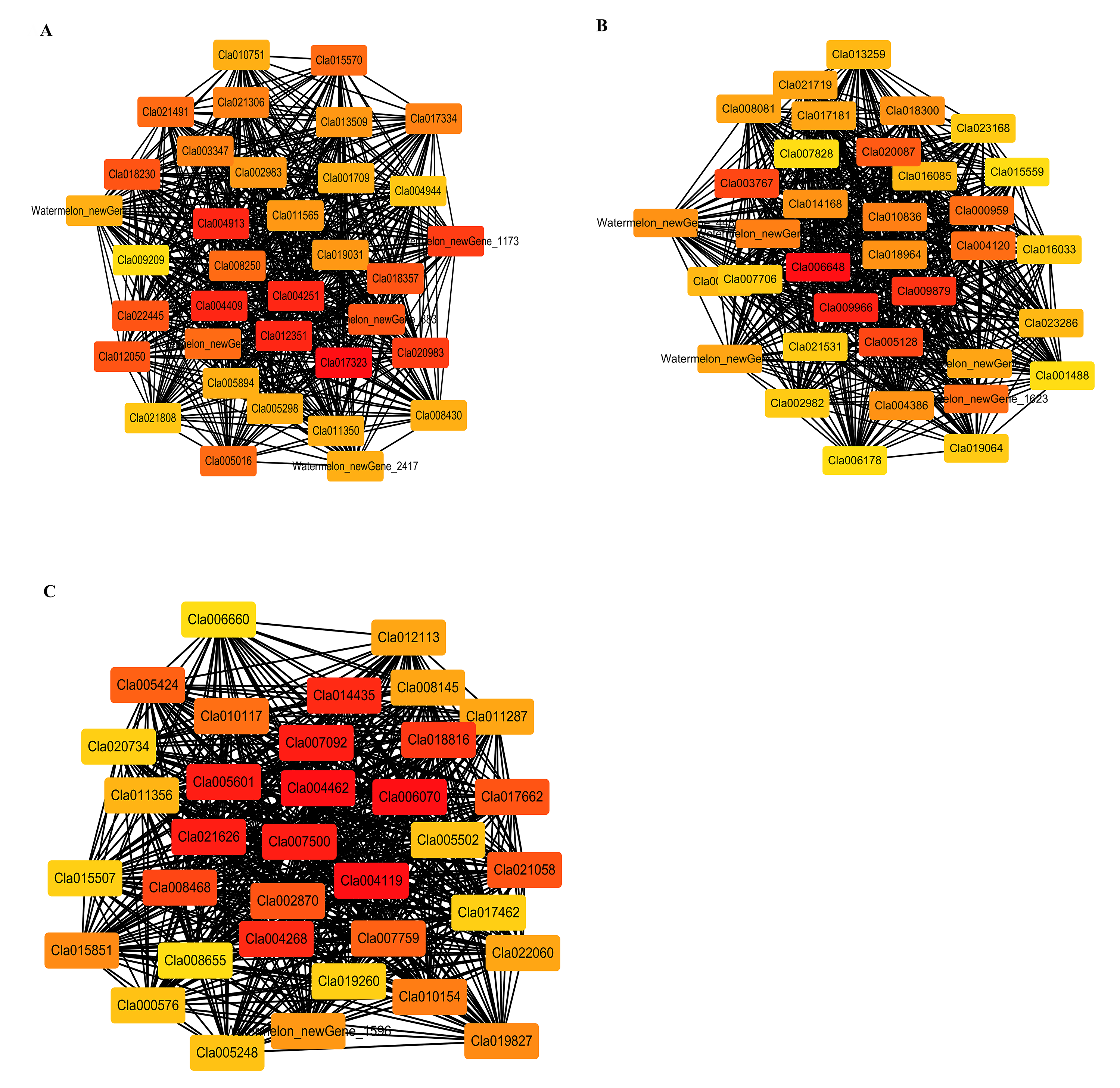

Supplement: Supplementary Figure 4 — Network visualization of hub genes (A) Dark-green module gene, (B) light-cyan (C) grey60 module gene. The red color heightened cells indicate the hub-genes. [file Image_4.TIF]

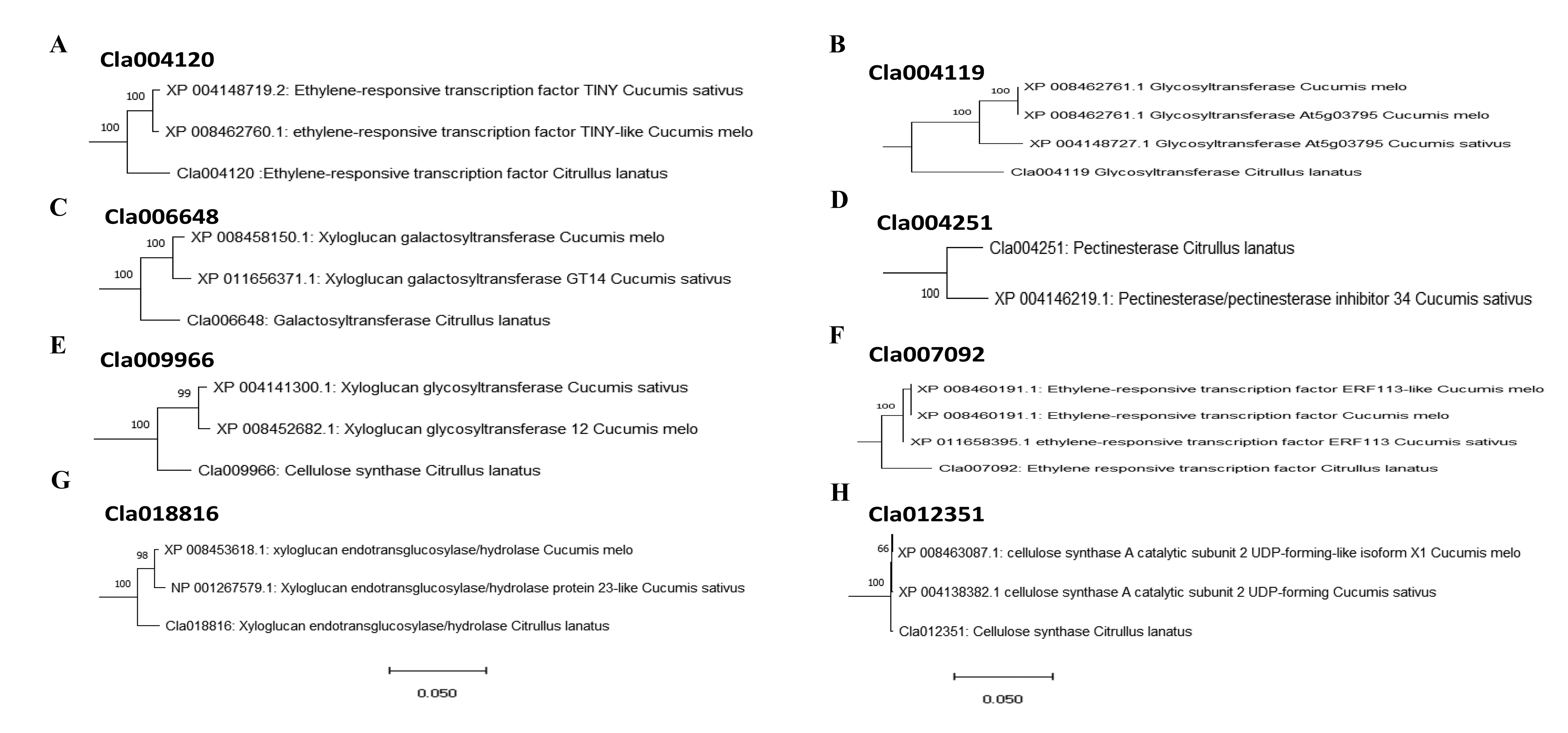

Supplement: Supplementary Figure 5 — Phylogeny of watermelon genes involved in regulation and transport of flesh firmness. The phylogenetic tree was created from protein sequences by maximum likelihood method. [file Image_5.TIF]
