# Supplementary material for: Identification of Key Gene Networks Associated With Cell Wall Components Leading to Flesh Firmness in Watermelon
Source: Front Plant Sci. 2021 Jun 22;12:630243. doi: 10.3389/fpls.2021.630243 (PMC8259604; doi:10.3389/fpls.2021.630243)
Supplement: Supplementary Table 1 — Primer used for q-RT-PCR. [file Data_Sheet_1.zip › Data sheet 1/Supplementary Tables 1, 2 and 4.pdf]

Identification of key gene-networks associated with cell wall components leading to Flesh-firmness in watermelon.

Muhammad Anees[1†], Lei Gao[1†], Muhammad Jawad Umer[1], Pingli Yuan[1], Hongju Zhu[1], Xuqiang Lu[1], Nan He[1], Chengsheng Gong1, Kaseb M O[1], Shengjie Zhao [1*] and Wenge Liu[1*]

1. Zhengzhou Fruit Research Institute, Chinese Academy of Agricultural Sciences, Henan Joint International Research Laboratory of South Asian Fruits and Cucurbits, Zhengzhou, P.R. China.

Supplementary Table 1 Primer used for q-RT-PCR.

Primer name	Forward Primer	Revers Primer
Cla012351	GGCTCGAGCGTTTCTCATAC	TTGCTTAGCTCAGGGACGAT
Cla007092	CAAAGACCATGGGGAAAATG	GCGGCGTTATCATAAGCAAT
Cla004120	TCCTCCTCCTCCTCTTCCTC	AAACTCGGGAGTTCCAGGAT
Cla004119	AGCACTTTGCAATGCTGATG	GCAAGAATGTGCCTCTGTGA
Cla009966	GTGGAGGATCAAAGCTCTCG	GATGAGCACGAACAGCGATA
Cla004251	CAAGACACGTGTACGGATGG	CGGAATTATCCTCGTCGTGT
Cla006648	GGCATTTGCCTGAGAATCAT	TCATGGCGGATTCTCTATCC
Cla018816	GACCGATTGGTCTCAAGCTC	AGGATGACTGTCCCGATGAC

Supplementary Table 2 Comprehensive Evaluation of RNA-Seq Data: A: 203Z, B: HWF. A Total Mapped Reads (%) = Unique Match (%) + Multi-position Match (%), are the percentages of clean reads align to reference genome. b Q30 (%) are the percentages of reads with Phred qualities scores over than 30**.**

Sample	Raw reads number	Clean reads number	Total mapped reads (%)	Unique match (%)	Multi-position match (%) [a]	GC content (%)	Q30(%)
203Z-10DAP	252449	246744	82.84	78.95	3.89	45.02	92.00
203Z-10DAP	245736	239813	82.11	79.30	2.80	44.69	91.60
203Z-10DAP	261051	255334	82.51	79.87	2.63	44.46	91.72
HWF-10DAP	269872	262073	83.91	80.91	3.00	44.34	92.05
HWF10DAP	261916	254792	82.94	80.07	2.87	44.66	91.75
HWF-10DAP	280677	272313	81.82	77.32	4.50	45.12	91.38
203Z-18DAP	281676	274662	82.85	78.75	4.10	44.91	92.27
203Z-18DAP	263276	257037	82.62	79.05	3.57	44.93	92.33
203Z-A8DAP	234342	228038	82.72	80.75	1.97	44.27	92.47
HWF-18DAP	375406	366735	84.27	80.12	4.15	44.79	93.52
HWF-18DAP	315724	309378	85.59	83.91	1.68	44.28	93.89
HWF-18DAP	341955	331472	86.33	84.47	1.86	43.81	93.55
203Z-26DAP	273710	267607	83.83	80.09	3.74	45.13	92.89
203Z-26DAP	296057	290491	83.24	80.51	2.73	44.65	92.60
203Z-26DAP	312683	306398	83.06	79.95	3.11	44.88	92.45
HWF-26DAP	282064	276564	83.42	81.11	2.31	44.71	92.12
HWF-26DAP	252242	245054	83.42	81.42	2.00	44.79	91.78
HWF-26DAP	283633	277195	82.23	79.26	2.97	44.88	91.46
203Z-34DAP	269347	262963	82.30	79.56	2.74	44.56	91.35
203Z-34DAP	270914	264168	82.50	79.32	3.18	44.73	91.74
203Z-34DAP	319919	311281	82.19	78.98	3.22	44.70	92.35
HWF34DAP	319673	312480	83.16	80.94	2.22	44.59	91.62
HWF-34DAP	318840	311985	83.04	80.50	2.53	44.58	91.66
HWF-34DAP	302566	294699	82.90	80.29	2.61	44.64	91.32

Supplementary Table 4 identified QTLS for fruit flesh firmness in different crops.

Crop	Chromosome/QTL	Reference
Melon	chr6,8,9,11 and 12	(Nimmakayala et al., 2016)
Apple	chr10	(Fabrizio et al., 2010)
Tomato	chr2	(N. H. Chapman et al., 2012)
Sweet cherry	qP-FF4.1	(Lichun et al., 2019)
